# SpatialLeiden: spatially aware Leiden clustering

**DOI:** 10.1186/s13059-025-03489-7

**Published:** 2025-02-07

**Authors:** Niklas Müller-Bötticher, Shashwat Sahay, Roland Eils, Naveed Ishaque

**Affiliations:** 1 Berlin Institute of Health at Charité – Universitätsmedizin Berlin, Center of Digital Health, Charitéplatz 1, 10117 Berlin, Germany; 2 Department of Mathematics and Computer Science, Freie Universität Berlin, Arnimallee 14, 14195 Berlin, Germany; 3 Health Data Science Unit, Heidelberg University Hospital and BioQuant, University of Heidelberg, Heidelberg, Germany

**Keywords:** Spatial omics, Clustering, Leiden, Domains, Niches, Spatial clustering, Spatial biology, Bioinformatics

## Abstract

Clustering can identify the natural structure that is inherent to measured data. For single-cell omics, clustering finds cells with similar molecular phenotype after which cell types are annotated. Leiden clustering is one of the algorithms of choice in the single-cell community. In the field of spatial omics, Leiden is often categorized as a “non-spatial” clustering method. However, we show that by integrating spatial information at various steps Leiden clustering is rendered into a computationally highly performant, spatially aware clustering method that compares well with state-of-the art spatial clustering algorithms.

**Supplementary Information:**

The online version contains supplementary material available at 10.1186/s13059-025-03489-7.

## Background

Single-cell transcriptomics has revolutionized our understanding of cellular heterogeneity by enabling the measurement of gene expression at the individual cell level. However, this high-dimensional data poses significant challenges in extracting meaningful biological insights. This can be overcome by grouping cells with similar expression profiles into distinct clusters. By partitioning cells based on transcriptional similarities, clustering facilitates the characterization of cell-type diversity within a heterogeneous cell population. Furthermore, clustering provides a basis for downstream analyses, such as differential expression, trajectory inference, and cell–cell interaction. In single-cell transcriptomics, a variety of clustering algorithms have been used and Leiden clustering has emerged as a performant choice [1]. Leiden clustering can be extended to consider multiomics data via the Leiden multiplex functionality [2].

Progress in spatially resolved omics methods has empowered researchers with the ability to map gene expression in a spatial manner, transcending conventional cell clustering approaches [3]. With spatial omics, scientists can discern higher-order tissue structures, termed spatial domains, by integrating spatial information alongside gene expression data. The identification of spatial domains through spatial clustering has emerged as a standard practice in constructing spatial atlases. This is instrumental in visualizing tissue anatomy, delineating tissue spatial continuity, pinpointing domain-specific marker genes, and unravelling domain-dependent molecular regulatory networks. Performance of spatial domain identification improves when leveraging the spatial information compared to non-spatial methods [4].

The Leiden algorithm clusters nodes in a network by optimizing a quality function, in a simple case this can be the modularity, which maximizes the differences between the actual number of edges in a community and the expected number of such edges under a null model. In single-cell transcriptomics, the cells (nodes) are connected to other cells based on the distance between cells in the gene expression space (edges), usually in a dimensionality reduced latent space. Leiden clustering therefore has been typically categorized as a “non-spatial” clustering method. However, spatial information can be leveraged at various processing steps, e.g., through spatial feature engineering or directly in the clustering procedure rendering Leiden spatially aware. Previous work in the domain of geographic health care service areas extended Leiden by integrating spatial information and enforcing spatially contiguous clusters [5]. However, for spatial transcriptomics this approach seems less suitable as domains can be spatially separated, e.g., in repeating or symmetric structures such as the two brain halves. Another approach is to include spatial embeddings using Leiden multiplex. Leiden multiplex enables the user to define an arbitrary number of networks (layers) with the same set of nodes (cells or spots) that describe different modalities of edges between the nodes. So, in spatially resolved omics data, the spatial neighborhood can be encoded by defining a spatial connectivity (based on, e.g., Euclidean distance) as the weight of edges between nodes as an additional layer to the typical *k*-nearest neighbors in gene expression space.

## Results and discussion

**Fig. 1:**
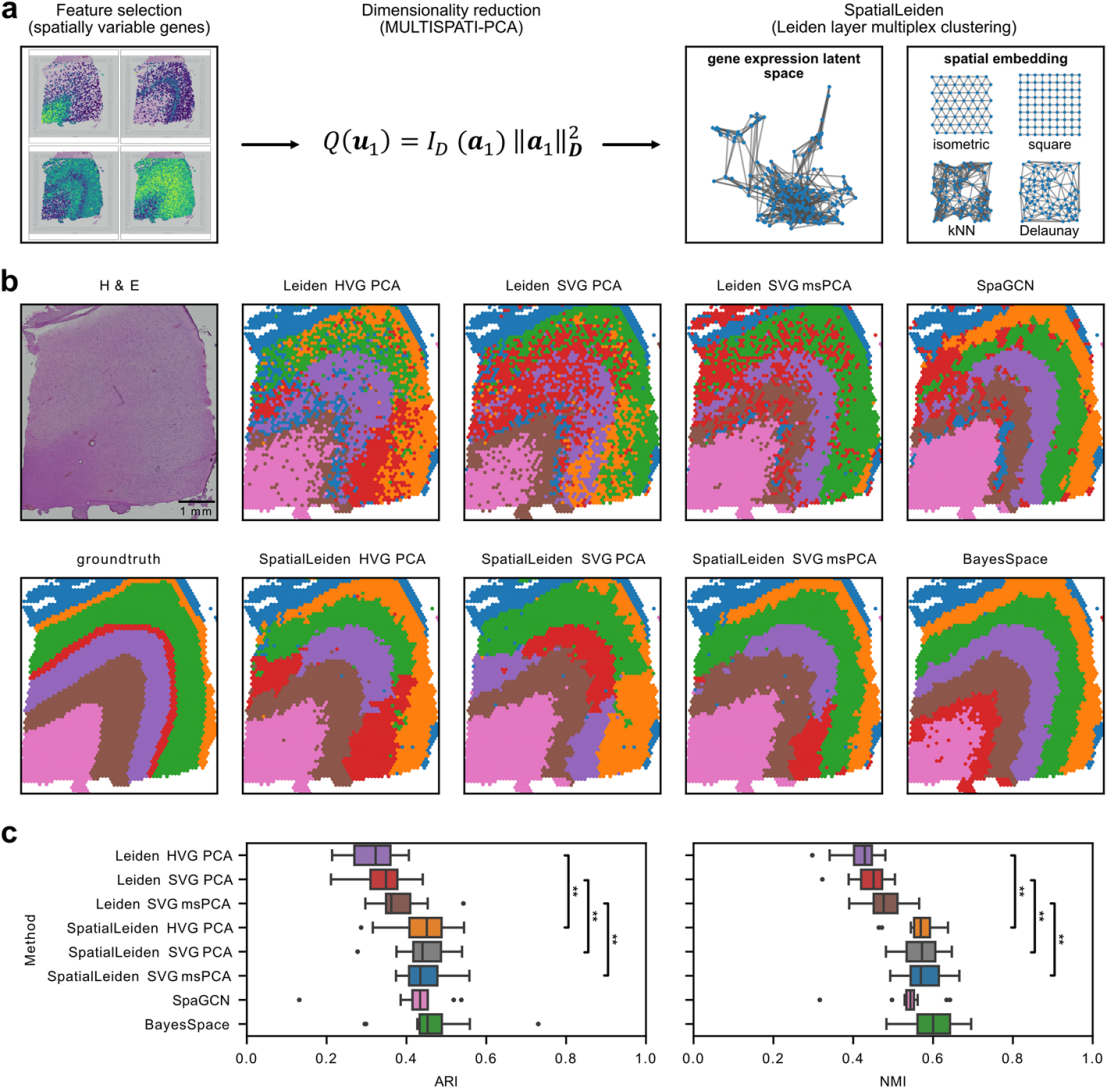
SpatialLeiden workflow. **a** Schematic of data processing and modelling steps to enable spatially aware Leiden clustering; feature selection can be performed by, e.g., SVG selection, dimensionality reduction by, e.g., MULTISPATI-PCA, and clustering is performed by SpatialLeiden. **b** Histology and manually annotated neocortex layered domains for the mouse brain DLPFC (slice 151673) and spatial domains detected by Leiden, SpatialLeiden, SpaGCN, and BayesSpace. **c** Box plot of ARI and NMI for all 12 DLPFC samples. Performance of SpaGCN and BayesSpace is in line with previous reports [11]. Center line: median; box limits: upper and lower quartiles; whiskers: 1.5 × interquartile range; dots: outliers; asterisks: significance (false discovery rate, two-sided Wilcoxon signed-rank test), only shown for Leiden vs corresponding SpatialLeiden (see Additional File 1: Table S1, S2)

In this study, we review how Leiden clustering can utilize spatial information through selection of spatially variable genes (SVGs) instead of highly variable genes (HVGs) [6], spatially aware dimensionality reduction through MUTLISPATI-PCA [7] (msPCA), and explicitly modelling the spatial embedding in the Leiden multiplex clustering (SpatialLeiden) (Fig. 1a). We demonstrate their application to a 10x Visium spatial transcriptomics dataset of the human dorsolateral prefrontal cortex (DLPFC), the most widely used benchmark dataset for spatial clustering methods [8]. This dataset consists of spatial gene expression data and histology images of 4 replicate slices from 3 donors, together with ground truth annotation of anatomical domains in those tissue samples. We compare the performance of a non-spatially aware and SpatialLeiden clustering to two widely used spatially aware domain detection tools, SpaGCN and BayesSpace [9, 10], and evaluate the performance of the tools (Fig. 1b, c).

While use of SVGs over HVGs yielded only minor improvements, we observed substantial improvement in performance when using spatially aware dimensionality reduction (msPCA) and using SpatialLeiden over non-spatial Leiden, revealing a better representation of the neocortex layering pattern (Fig. 1b, Additional File 1: Fig. S1-S3). We quantitatively evaluated performance of the different clustering strategies using the Adjusted Rand Index (ARI) and Normalized Mutual Information (NMI) score, showing significant improvements of SpatialLeiden over the non-spatial Leiden implementation (Additional File 1: Table S1, S2), with performance that was comparable to SpaGCN and BayesSpace (Fig. 1c) at a fraction of the processing time. SpatialLeiden performed favorably when we compared its performance to other tools in a recent benchmark study, ranking 5th of 15 tools [4] (see Additional File 1: Supplementary Methods for further details).

As with other multi-modal clustering approaches, careful consideration has to be paid to a number of parameters including the resolution to be applied to each modality (with the same implications as in Leiden, i.e., a higher resolution meaning stronger within cluster connectivity and therefore more clusters) and the weight of each modality (Additional File 1: Fig. S4). Furthermore, the spatial relationship between cells can be modelled in different ways; a regular grid pattern is suitable for Visium (isometric) and binned Stereo-seq (square), while for imaging-based spatially resolved transcriptomics methods Delaunay triangulation or *k*-nearest neighbors can be used to define the spatial layer. We investigated the effect of the neighborhood structure and size and found it to have similar effects as changing the weighting of the spatial layers (Additional File 1: Fig. S5, S6).

Many domain identification methods integrate spatial and gene expression information into a joint latent space, which is then clustered using conventional so-called “non-spatial” clustering methods, such as Leiden. To investigate whether using SpatialLeiden can further enhance domain identification, we generated spatially informed latent spaces using Banksy [12] and SpiceMix [13] while varying the influence of the spatial information in the latent space. SpatialLeiden improved domain identification, particularly when the spatial information contributed less to the joint latent space (Additional File 1: Fig. S7).

**Fig. 2:**
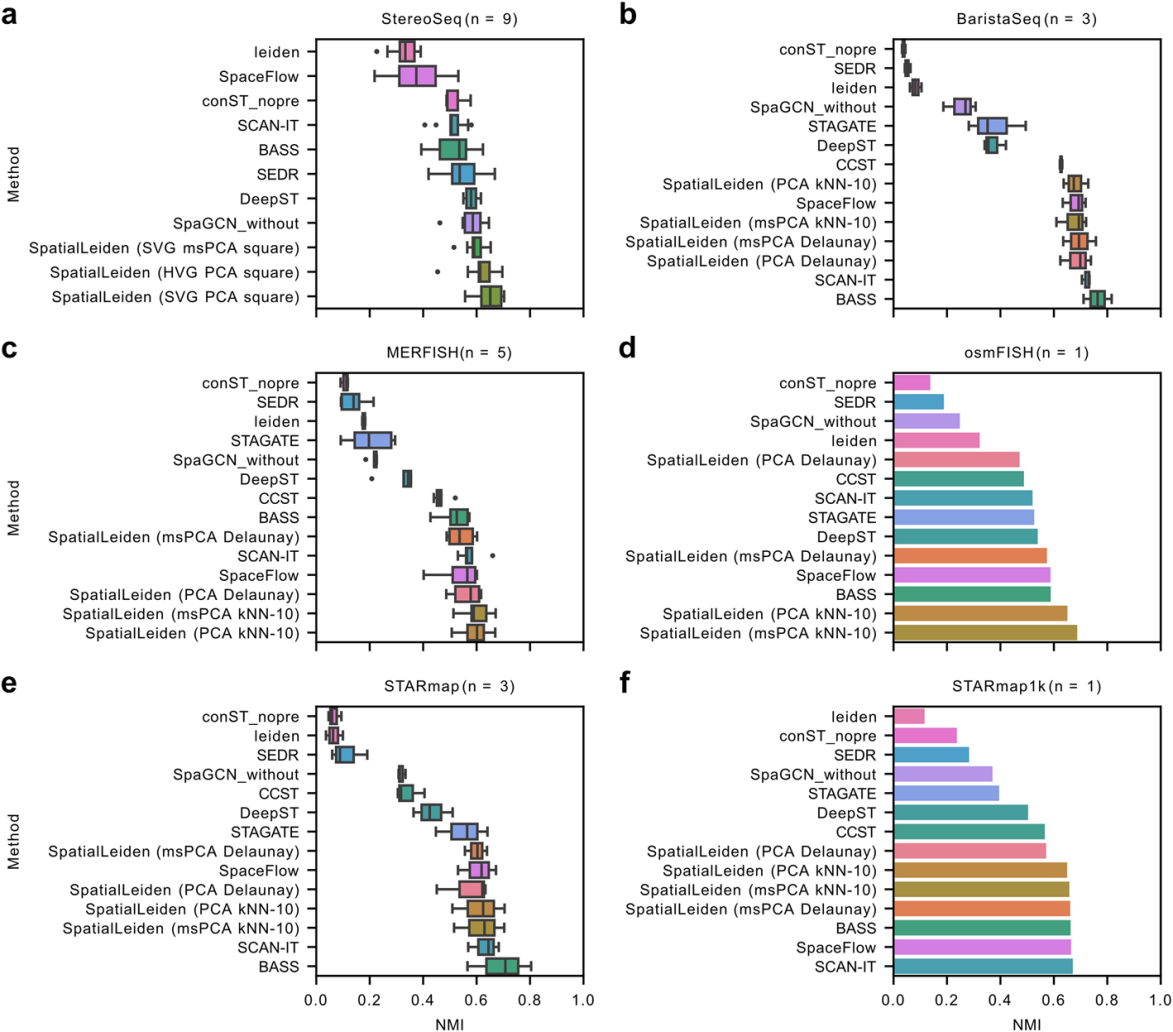
Performance of SpatialLeiden across technologies and tissues. **a** Stereo-Seq of the mouse embryo at various development stages. **b** BaristaSeq of mouse primary cortex. **c** MERFISH mouse brain hypothalamus preoptic area. **d** osmFISH of mouse somatosensory cortex. **e** STARmap mouse brain medial prefrontal cortex. **f** STARmap* of mouse visual cortex. Performance metrics of other tools are taken from Yuan et al. [4]. Methods were run with 5 different random seeds and median results were reported per sample. **a**–**c**, **e** Box plot of the median NMI with center line: median; box limits: upper and lower quartiles; whiskers: 1.5 × interquartile range; dots: outliers. **d**, **f** Bar plot of the median NMI

To test whether SpatialLeiden is applicable across technologies, tissues, and neighborhood models, we analyzed a number of datasets (Stereo-Seq mouse embryo [14], BaristaSeq mouse brain primary cortex, MERFISH mouse brain hypothalamus preoptic area [15], osmFISH mouse brain somatosensory cortex, STARmap mouse brain medial prefrontal cortex [16, 17], STARmap1k mouse brain visual cortex) and demonstrated exceptional improvements over non-spatially aware Leiden clustering. SpatialLeiden demonstrated top tier performance for all datasets (Fig. 2, Additional File 1: Table S3). For imaging-based spatial transcriptomics methods, we found that modelling the spatial neighborhood using the 10 *k*-nearest neighbors generally performed better than using Delaunay triangulation. When using HVG selection and PCA all non-Stereo-seq dataset were processed within 2.5 min utilizing less than 400 MB of RAM, and all Stereo-seq samples were processed in less than 8 min with a maximum of 2 GB of RAM (Additional File 1: Fig. S8).

## Conclusions

Our results show that the reference implementation of the Leiden algorithm can indeed be used as a spatially aware clustering algorithm. Subsequent studies that compare spatially aware clustering algorithms should clearly state that they compare to non-spatial implementation of Leiden, rather than misclassifying Leiden as a non-spatial algorithm.

We describe the different steps at which spatial awareness can be introduced into the analysis, and our implementation allows easy parameterization of key considerations for modelling gene and spatial modalities. While many spatial domain clustering tools rely on spatially aware dimensionality reduction approaches, this is often followed by non-spatial clustering, and we demonstrated that these methods can improve with spatially aware clustering such as SpatialLeiden.

Due to the algorithmic similarity between Louvain and Leiden clustering, our approach is directly applicable to Louvain community detection. Furthermore, leveraging an additional graph layer for the spatial neighborhood has the potential to be transferred to other graph-based clustering methods.

The same way Leiden became the method of choice for clustering of single-cell data, we believe that SpatialLeiden will become the method of choice for spatial data owing to its efficiency, simplicity, and ease of integration into existing analysis workflows.

## Methods

### Data processing

Data was analyzed using python (v3.10.14), Scanpy [18] (v1.10.1), and Squidpy [19] (v1.4.1).

#### Spatial neighborhood graph generation

The neighbors of each cell were defined depending on the technology. For datasets with a regular grid, the neighbors were defined using squidpy.gr.spatial_neighbors with coord_type *‘grid’,* n_rings set to 1, and n_neighs set to 6 or 4 for Visium and Stereo-seq, respectively (unless otherwise specified). For all other datasets, the neighbors were defined either using Delaunay triangulation (squidpy.gr.spatial_neighbors with delaunay = True) or using the *k*-nearest neighbors (squidpy.gr.spatial_neighbors with coord_type *'generic'* and n_neighs set to 10 unless otherwise specified). The untransformed neighborhood graph *‘spatial_connectivities’* was used as is for regular grids as all connections are equidistant. For Delaunay triangulation and *k*NN, the *‘spatial_distances’* were transformed to *‘spatial_connectivities’* via the following formula:

$$1-\frac{d}{{d}_{max}}$$

#### HVG and SVG detection

HVGs were detected based on filtered count data (scanpy.pp.highly_variable_genes with flavor *‘seurat_v3’*). To detect SVGs, first the neighbors were defined as described above, and the Moran’s *I* score was calculated for all genes using squidpy.gr.spatial_autocorr with mode *‘moran’* and selecting the top 3000 scoring genes. Gene selection was only performed for capture-based spatial transcriptomics technologies (Visium, Stereo-seq).

#### MULTISPATI-PCA

We implemented MULTISPATI-PCA [7] in python (https://github.com/HiDiHlabs/multiSPAETI , v0.1.0) and used it to perform spatially aware dimensionality reduction. The spatial neighborhood graph *‘spatial_connectivities’* was used to calculate 30 components (corresponding to the 30 largest eigenvalues) based on the 3000 HVGs/SVGs (Visium, Stereo-seq) or all genes in the case of imaging-based technologies (STARmap, STARmap*, MERFISH, BaristaSeq, osmFISH) as for normal PCA.

#### Latent neighborhood graph generation

The spatial neighborhood graph in the Visium grid *‘spatial_distances’* from squidpy.gr.spatial_neighbors (as described in SVG detection) is used as the spatial layer for Leiden clustering. To build the neighborhood graph in latent space of gene expression, we first calculated the first 30 principal components based on the top 3000 variable genes (scanpy.tl.pca). We identified the 15 nearest neighbors per spot (scanpy.pp.neighbors) based on the PCA or MULTISPATI-PCA results from either the HVGs/SVGs (Visium, Stereo-seq) or for all genes in the case of imaging-based technologies (STARmap, STARmap*, MERFISH, BaristaSeq, osmFISH) and used the resulting *‘connectivities’*.

#### Non-spatially aware Leiden

We used Leiden [1] (https://github.com/vtraag/leidenalg, v0.10.2) as implemented in Scanpy with the default parameters and varied the resolution to achieve the correct number of clusters for each of the DLPFC datasets following the approach of the SpaGCN.search_res function (https://github.com/jianhuupenn/SpaGCN).

### Spatially aware Leiden multiplex (SpatialLeiden)

We implemented a spatially aware version of Leiden (https://github.com/HiDiHlabs/SpatialLeiden, v0.1.0) by using the Layer multiplex [20]. An additional graph encoding for the spatial neighborhood of the cells was added as second layer in addition to the layer encoding gene expression in latent space. The additional spatial layer was encoded as RBConfigurationVertexPartition as is the default for the scanpy implementation for the latent space graph. The optimal clustering was identified by running the Optimiser.optimise_partition_multiplex from leidenalg until convergence. As only the ratio of the layer weights is relevant, the weight for the gene expression latent space layer was kept at 1, and the weight for the spatial neighborhood was set depending on technology and method of neighborhood definition. The resolution for the latent space partition was set by running the standard Leiden clustering and identifying the resolution which yields the correct number of clusters. The resolution of the spatial partition was then varied to identify the correct number of clusters in the multiplex Leiden using the same approach as described for the standard Leiden method.

### Implementation and comparison to other spatial clustering algorithms

Implementation and comparison to other spatial clustering algorithms is described in the supplemental methods.

### Statistical testing

To test significant differences in ARI or NMI, two-sided Wilcoxon signed-rank tests were performed and the false discovery rate correction calculated with the Benjamini–Hochberg procedure.

## Supplementary Information

Additional File 1: Supplementary Information. Supplementary methods, Supplementary Figures (Fig. S1-S8), and Supplementary Tables (Table S1-S4).

Additional File 2: Source data. Data to reproduce Fig. 1c, 2 , and S8.

## Data Availability

This study used publicly available spatially resolved transcriptomics data of the mouse brain DLPFC profiled on the 10x Visium platform (http://research.libd.org/spatialLIBD/). Public Stereo-seq, STARmap, STARmap*, MERFISH, osmFISH, and BaristaSeq datasets were downloaded from http://sdmbench.drai.cn/. Performance metrics of other tools presented in Fig. 2 were taken from Yuan et al. [4]. We provide a repository with the code for reproducing all results and figures of this study https://github.com/HiDiHlabs/SpatialLeiden-Study. The two python packages implemented as part of this study are publicly available on GitHub; multispaeti (https://github.com/HiDiHlabs/multiSPAETI) and spatialleiden (https://github.com/HiDiHlabs/SpatialLeiden). The versions used in this manuscript are deposited on zenodo; multispaeti (10.5281/zenodo.14724796)[21] and spatialleiden (10.5281/zenodo.14724819) [22]. All code is free and open source and licensed with an MIT license.
