# Supplementary material for: SpatialLeiden: spatially aware Leiden clustering
Source: Genome Biol. 2025 Feb 7;26:24. doi: 10.1186/s13059-025-03489-7 (PMC11804054; doi:10.1186/s13059-025-03489-7)
Supplement: Supplementary file 1 — Additional File 1: Supplementary Information. Supplementary methods, Supplementary Figures (Fig. S1-S8), and Supplementary Tables (Table S1-S4). [file 13059_2025_3489_MOESM1_ESM.docx]

Supplementary Information

Supplementary information for “SpatialLeiden: spatially-aware Leiden clustering” by Niklas Müller-Bötticher, Shashwat Sahay, Roland Eils, and Naveed Ishaque.

Content

[Supplementary Methods 2](#_Toc188957948)

[Data processing 2](#_Toc188957949)

[BayesSpace 2](#_Toc188957950)

[SpaGCN 2](#_Toc188957951)

[Banksy 2](#_Toc188957952)

[SpiceMix 2](#_Toc188957953)

[ARI and NMI calculation 3](#_Toc188957954)

[Data analysis 3](#_Toc188957955)

[Runtime performance metrics 3](#_Toc188957956)

[Comparison to other benchmarking studies 4](#_Toc188957957)

[Supplementary Figures 5](#_Toc188957958)

[Supplementary Tables 13](#_Toc188957959)

# Supplementary Methods

## Data processing

Data was analyzed using python (v3.10.14), Scanpy (v1.10.1), and Squidpy (v1.4.1).

## BayesSpace

BayesSpace is a method that applies Bayesian statistics to a low-dimensional representation of gene expression for spatial clustering, optimizing for neighboring points to having the same cluster membership through a spatial prior. BayesSpace is one of the early and highly cited tools for spatial domain identification. We used BayesSpace (v1.10.1, R v4.3.1) using preprocessing and parameters as recommended in the original publication and package vignette. Our observed results (median ARI: 0.45) were not lower than the recently reported ARIs by Hu et al.[11] (0.37).

## SpaGCN

SpaGCN is a method that utilizes a graph convolutional neural network graph to integrates gene expression, spatial location and histology for domain identification. SpaGCN is another one of the early and highly cited tools for spatial domain identification. We used SpaGCN (v1.2.7, python v3.8.18) using preprocessing and parameters as defined in the original publication and package tutorial. Our observed results (median ARI: 0.43) were in line with the recently reported ARIs by Hu et al.[11] (0.41).

## Banksy

Banksy (v0.99.13, R v4.4.1) integrates spatial information by combining the expression of a cell with that of its local microenvironment and allows to control the influence of the microenvironment via a mixing parameter λ. The data was processed by identifying 2,000 highly variable genes (HVG) with Seurat (v5.1.0) using FindVariableFeatures after running NormalizeData, and normalizing the counts with scuttle’s (v1.16.0) normalizeCounts. The neighborhood components were computed using computeBanksy with k_geom set to 18 and compute_agf to true and 20 components were identified using runBanksyPCA with use_agf set to true and varying values for lambda. Clustering was then performed with Leiden and SpatialLeiden as described in the methods.

## SpiceMix

SpiceMix (popari v0.0.71) finds a latent representation by extending an NMF with the spatial location of cells. The data was preprocessed with scanpy (v1.10.3) by normalizing the counts per cell to 10,000 (scanpy.pp.normalize_total), log transforming the data (scanpy.pp.log1p), and detecting 3,000 HVGs (scanpy.pp.find_highly_variable). Spatial adjacency of cells was identified using popari.PopariDataset.compute_spatial_neighbors. The SpiceMix model was then trained to identify 20 factors using 5 pre-iterations and 200 iterations with varying spatial regularization parameter lambda_Sigma_x_inv. The resulting embedding was normalized with popari.preprocess_embeddings and clustered with Leiden and SpatialLeiden as described.

## ARI and NMI calculation

The Adjusted Rand Index (ARI) and Normalized Mutual Information (NMI) were calculated using the adjusted_rand_score and normalized_mutual_info_score functions in scikit-learn (v1.5.0) with all parameters at default values.

## Data analysis

### Capture-based technologies

Capture-based datasets (Visium human brain dorsolateral prefrontal cortex, Stereo-Seq mouse embryo) were preprocessed by removing genes that appear in less than 10 spots (scanpy.pp.filter_genes), normalizing the total count of each cell (scanpy.pp.normalize_total with target_sum 10,000), and log transforming the data (scanpy.pp.log1p). The layer weight ratio of spatial to latent space graph was set to 0.8 in SpatialLeiden unless indicated otherwise.

### Imaging-based technologies

Imaging-based datasets (MERFISH mouse brain hypothalamus preoptic area, STARmap mouse brain medial prefrontal cortex, STARmap* mouse visual cortex, BaristaSeq mouse primary cortex, osmFISH mouse somatosensory cortex) were log transformed (scanpy.pp.log1p). The layer weight ratio for SpatialLeiden for MERFISH was set to 1 and 1.8 for Delaunay triangulation and kNN (10 neighbors), respectively. STARmap: 1 and 1.6, STARmap*: 0.8 and 1.4, BaristaSeq: 1.2 and 1.8, osmFISH: 0.8 and 1.2.

## Runtime performance metrics

To calculate runtime performance metrics jobs were submitted using slurm sbatch to a single compute node (-N 1) with 8 CPUs (-n 8) with each node consisting of a Dell PowerEdge C6520 with 2x Intel Xeon Gold 6130 or 6252 @ 2.1 GHz. The metrics were calculated from the slurm job information (using *‘ElapsedRaw’* as wall time, *‘TotalCPU’* as CPU time, and *‘MaxRSS’* as maximum memory usage) for the entire workflow including data loading, preprocessing, dimensionality reduction, and clustering.

## Comparison to other benchmarking studies

We compared the obtained median NMI (0.58) for multiplex Leiden clustering using SVGs and MULTISPATI-PCA on the Visium DLPFC dataset (**Fig. 1,** **Table S1, S2**) to those in a study by Yuan et al.[4]. They reported the NMI across 10 replicate runs for each sample over 14 tools in their Source Data Figure 2. The median NMI per tool, ordered by NMI, were: Louvain 0.24; Leiden 0.25; SpaGCN (w/ H&E): 0.48; SpaceFlow 0.49; STAGATE 0.50; CCST 0.51; conST_nopre 0.52; SpaGCN (w/o H&E): 0.53; SEDR 0.53; stLearn 0.54; BayesSpace 0.60; SCAN-IT 0.61; BASS 0.61; DeepST 0.62.

# Supplementary Figures


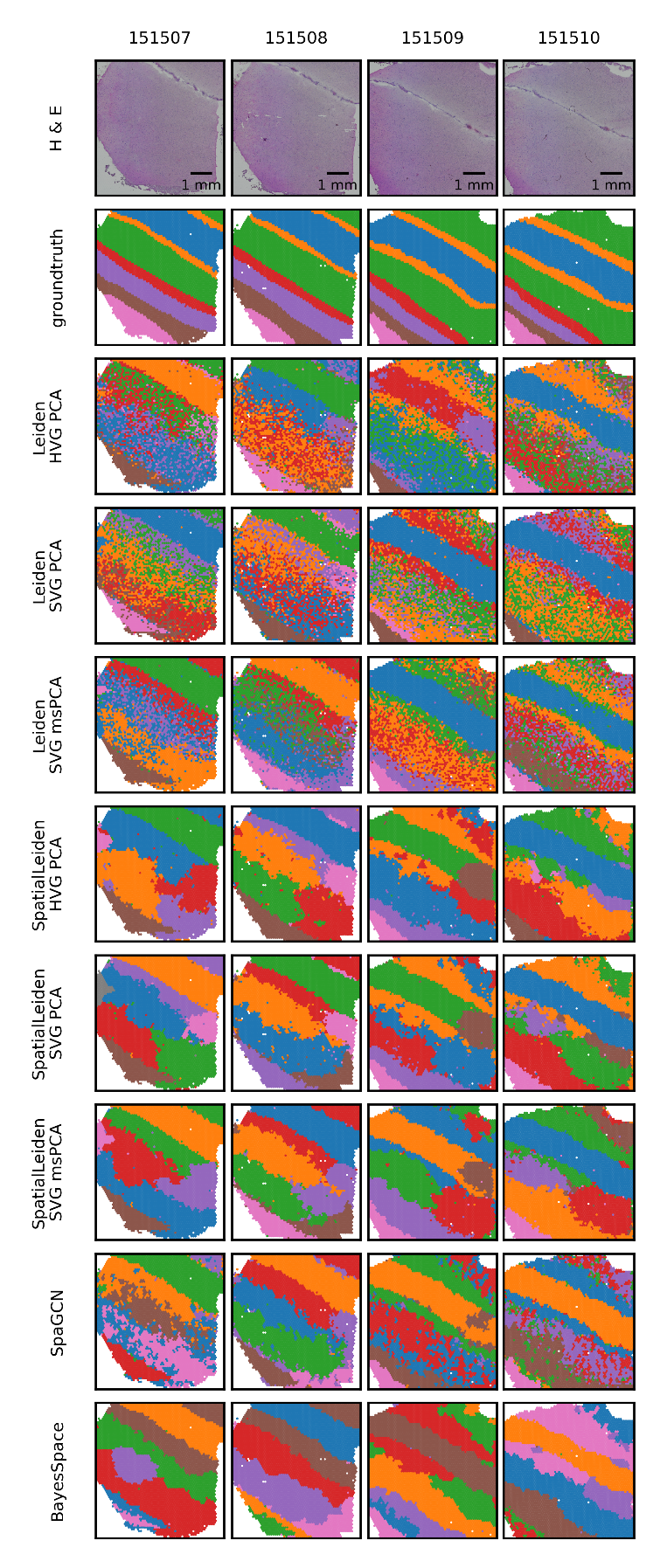


**Fig. S1**: Clustering results obtained from Visium samples of patient Br5292.


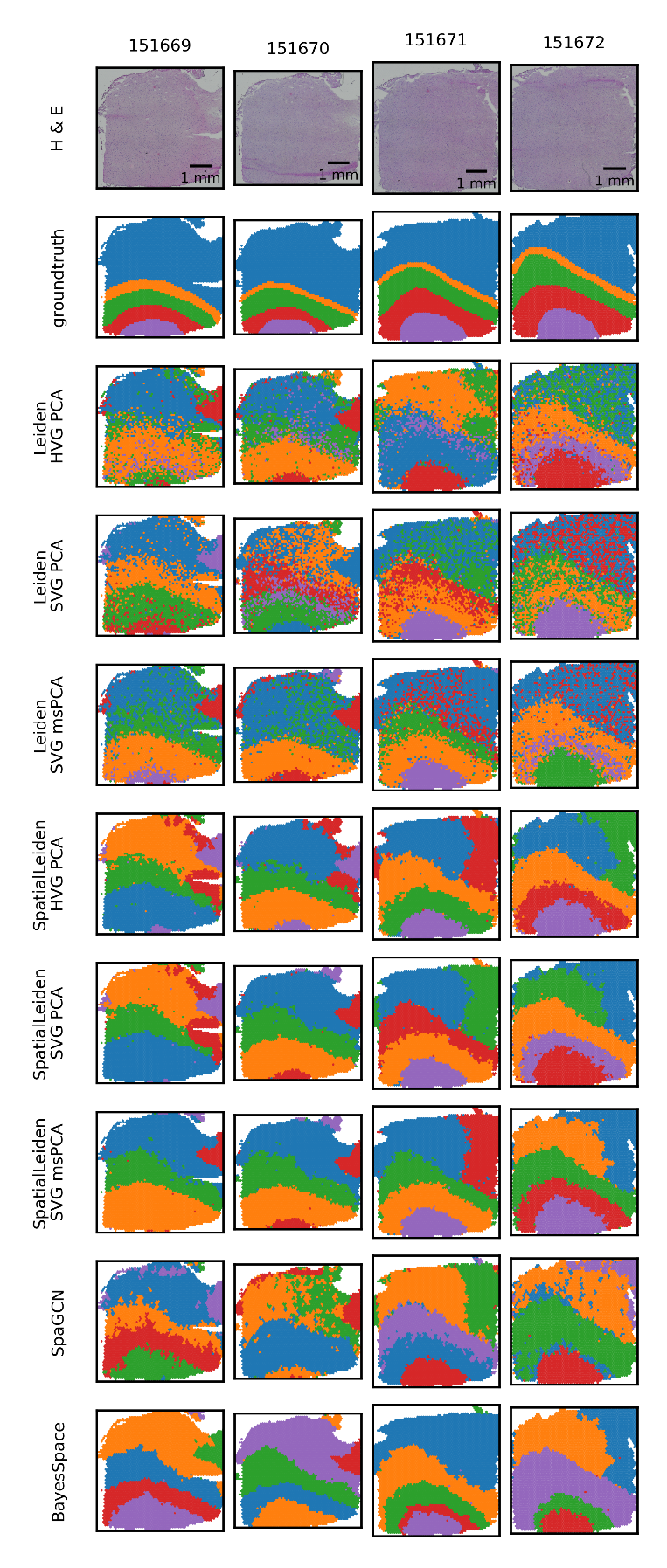
**Fig. S2**: Clustering results obtained from Visium samples of patient Br5295.

**
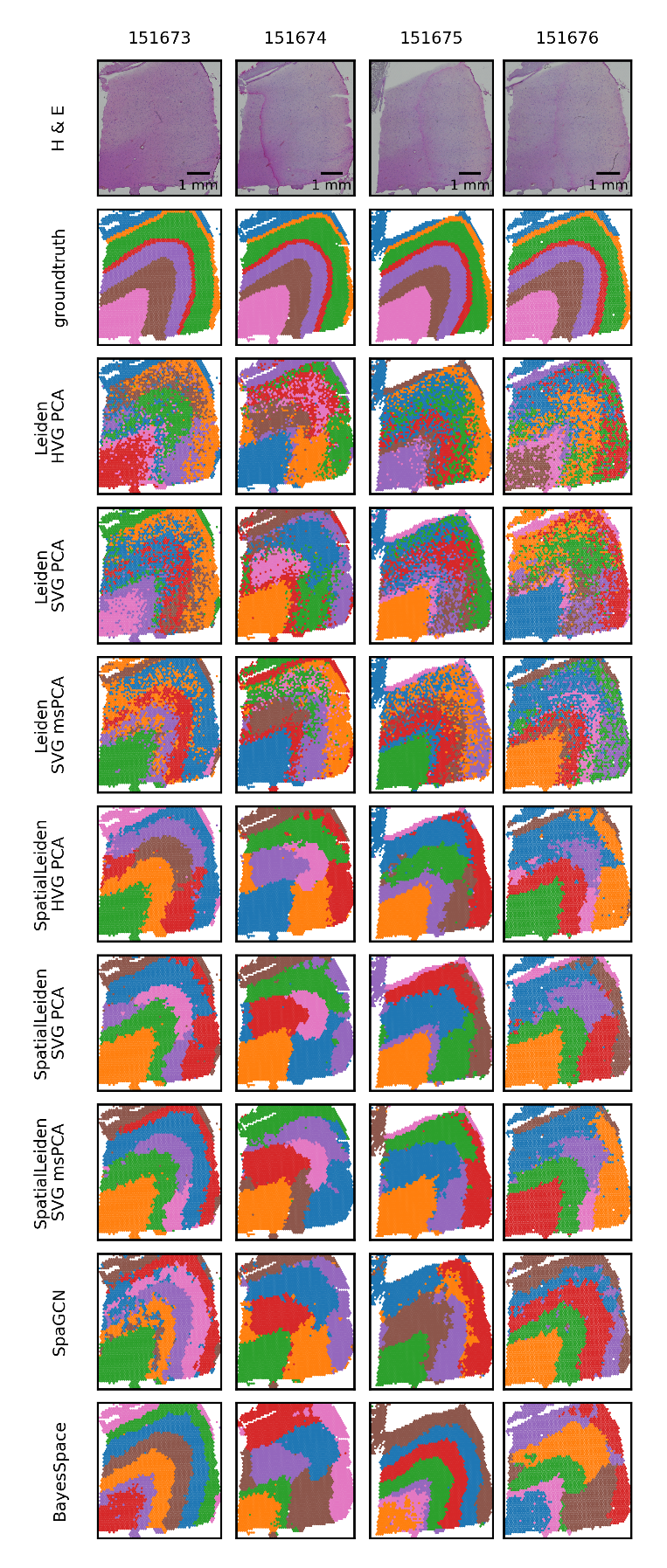
Fig. S3**: Clustering results obtained from Visium samples of patient Br8100.


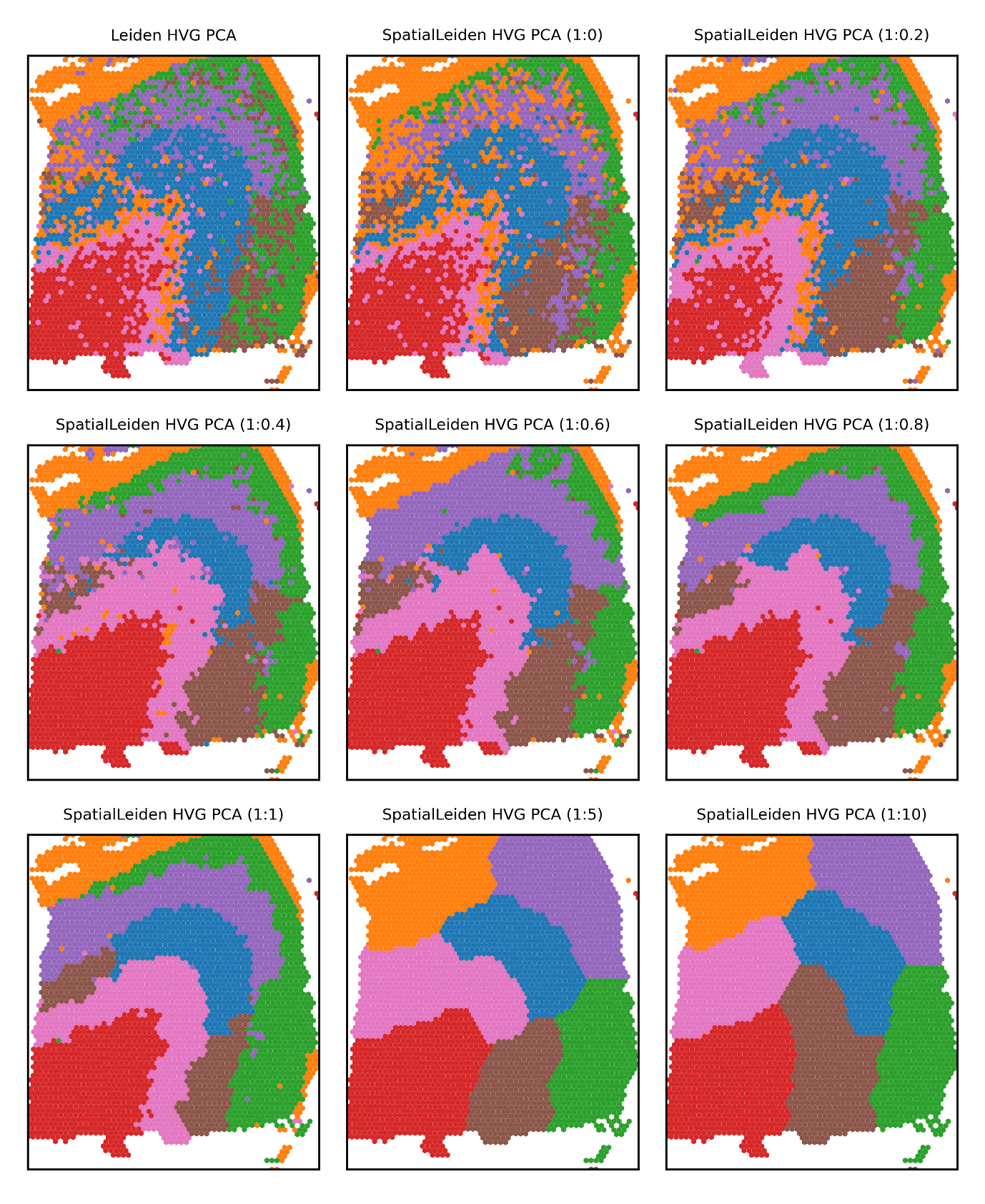
**Fig. S4**: The effect of weighting the gene expression vs spatial layers. Shown are the clustering results for Visium sample Br8100**-**151673 using Leiden and SpatialLeiden. The ratio of weights (expression:spatial) is shown in parenthesis in the subplot titles.


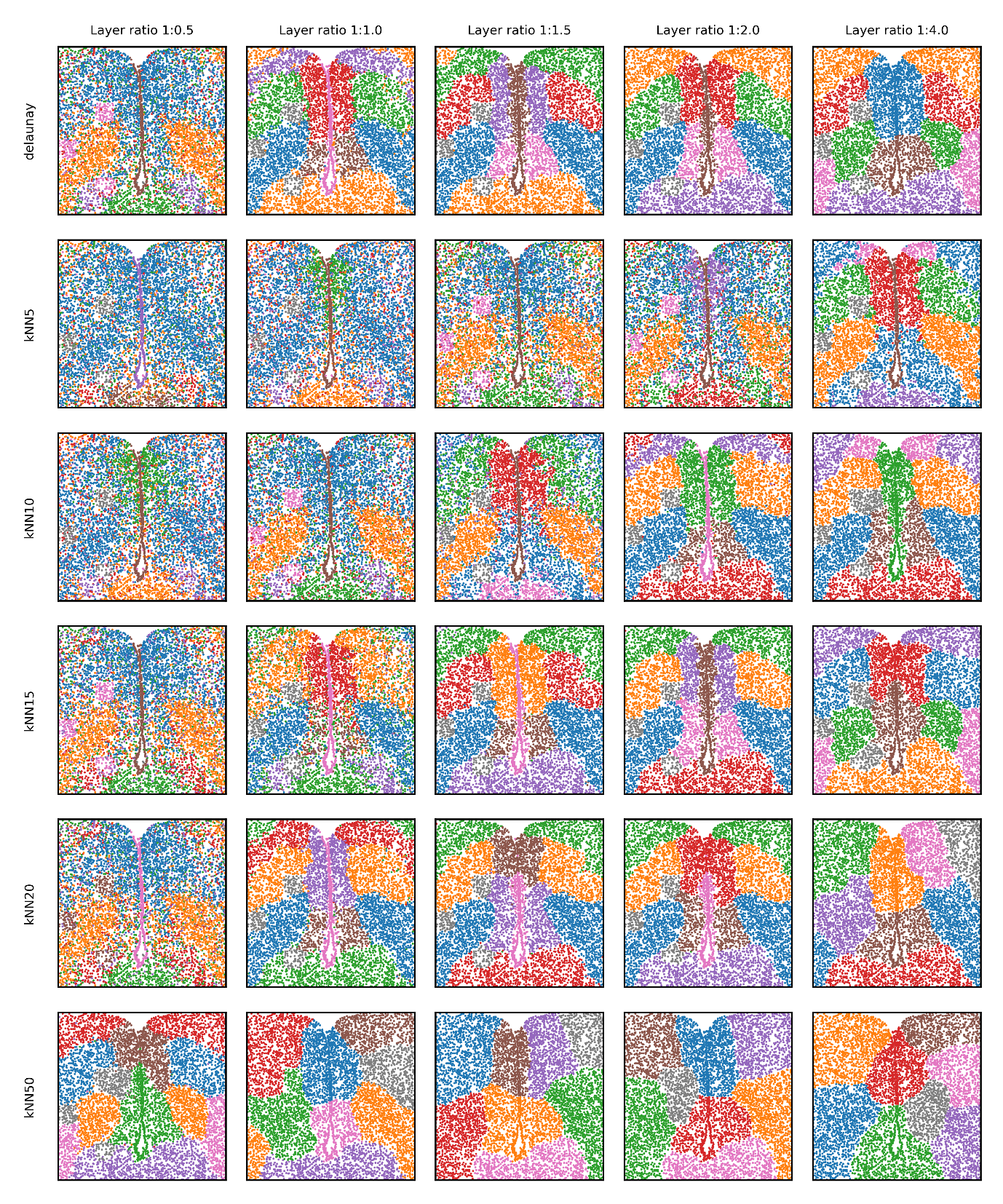
**Fig. S5**: The effect of varying neighborhood structure and size on MERFISH data. Shown are the clustering results for sample MERFISH_0.14 with the neighborhood identified as Delaunay triangulation or kNN (using different values for k) followed by clustering using Leiden and SpatialLeiden (layer weight ratio indicated as expression:spatial).

**
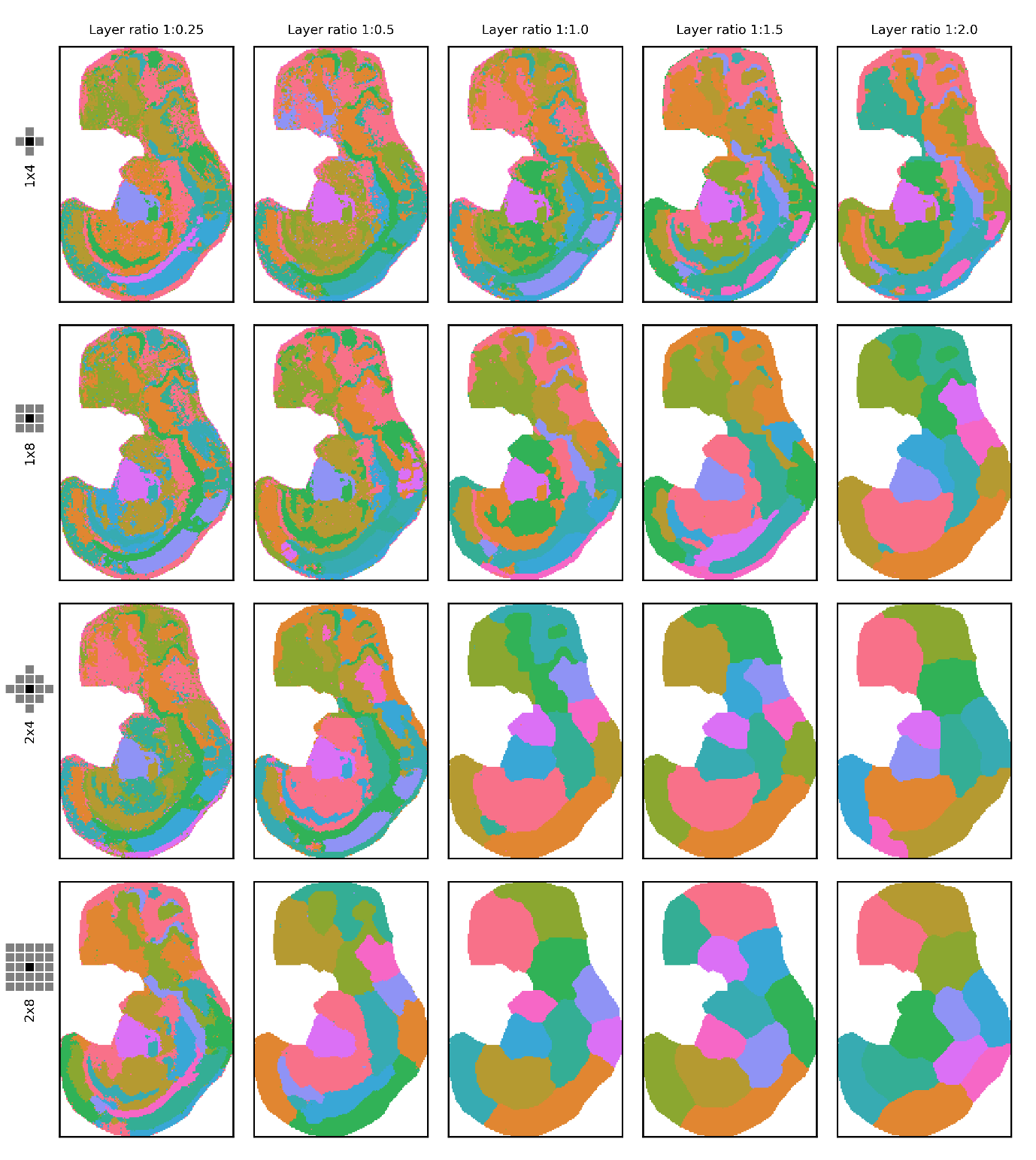
Fig. S6**: The effect of varying neighborhood size on Stereo-seq data. Shown are the clustering results for sample E10.5_E1S2.MOSTA with the neighborhood identified as 1 or 2 rings with 4 or 8 neighbors followed by clustering using Leiden and SpatialLeiden (layer weight ratio indicated as expression:spatial).


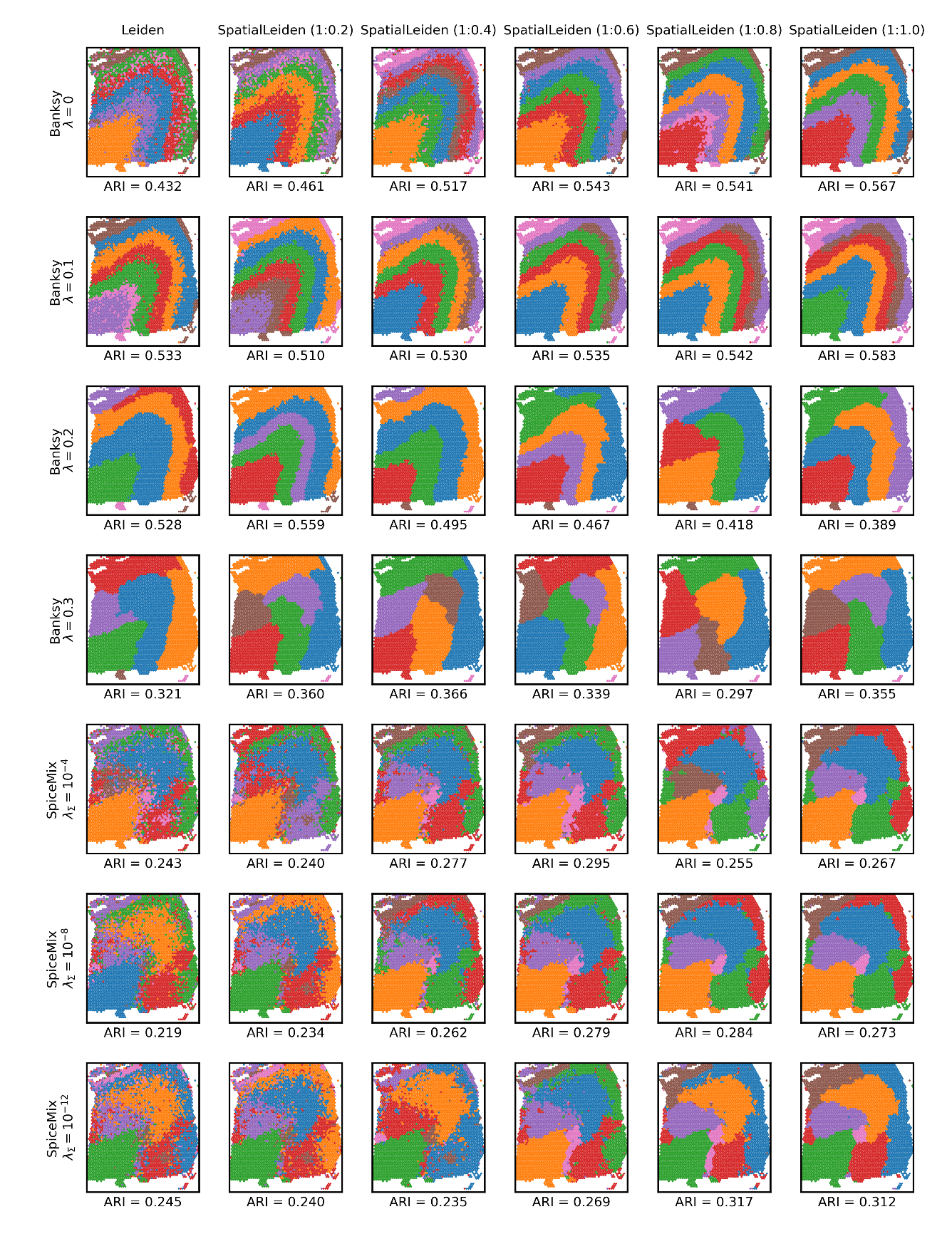
**Fig. S7**: SpatialLeiden can replace non-spatial clustering for domain identification tools. Shown are the clustering results for Visium sample Br8100**-**151673 with latent space generated by Banksy or SpiceMix followed by clustering using Leiden and SpatialLeiden. The ratio of weights (expression:spatial) is shown in parenthesis in the subplot titles.


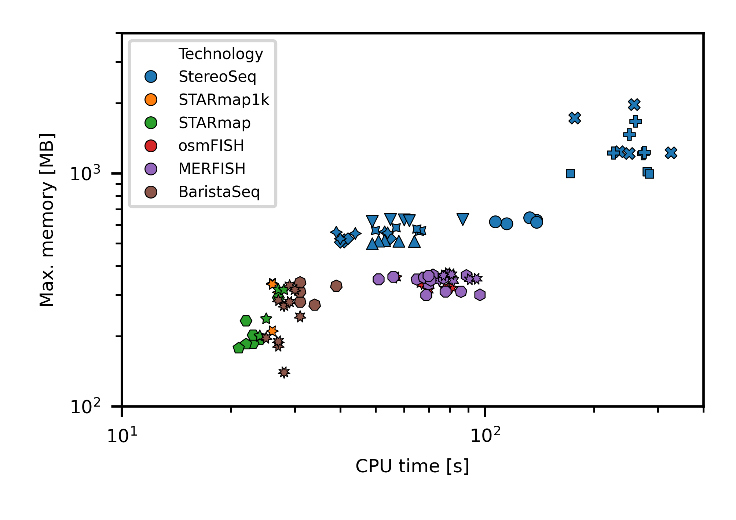
**Fig. S8**: Runtime statistics. Scatter plot of total CPU time vs max. memory used to process datasets across technologies. Colors represent the technology and the shape encodes the specific sample for each dataset. Each sample was processed 5 times with different starting seeds.

# Supplementary Tables

**Table S1:** Adjusted p-values for differences in ARI for the LIBD DLPFC dataset (two-sided Wilcoxon signed-rank test and Benjamini-Hochberg procedure).

| vs | BayesSpace | Leiden HVG PCA | Leiden SVG PCA | Leiden SVG msPCA | SpaGCN | SpatialLeiden HVG PCA | SpatialLeiden SVG PCA | SpatialLeiden SVG msPCA |
| --- | --- | --- | --- | --- | --- | --- | --- | --- |
| BayesSpace |  | 0.0059 | 0.0085 | 0.014 | 0.32 | 0.53 | 0.59 | 0.56 |
| Leiden HVG PCA | 0.0059 |  | 0.37 | 0.0046 | 0.014 | 0.0034 | 0.0034 | 0.0034 |
| Leiden SVG PCA | 0.0085 | 0.37 |  | 0.023 | 0.014 | 0.0096 | 0.0096 | 0.0034 |
| Leiden SVG msPCA | 0.014 | 0.0046 | 0.023 |  | 0.086 | 0.037 | 0.012 | 0.0046 |
| SpaGCN | 0.32 | 0.014 | 0.014 | 0.086 |  | 0.53 | 0.37 | 0.4 |
| SpatialLeiden HVG PCA | 0.53 | 0.0034 | 0.0096 | 0.037 | 0.53 |  | 0.73 | 0.43 |
| SpatialLeiden SVG PCA | 0.59 | 0.0034 | 0.0096 | 0.012 | 0.37 | 0.73 |  | 0.52 |
| SpatialLeiden SVG msPCA | 0.56 | 0.0034 | 0.0034 | 0.0046 | 0.4 | 0.43 | 0.52 |  |

**Table S2:** Adjusted p-values for differences in NMI for the LIBD DLPFC dataset (two-sided Wilcoxon signed-rank test and Benjamini-Hochberg procedure).

| vs | BayesSpace | Leiden HVG PCA | Leiden SVG PCA | Leiden SVG msPCA | SpaGCN | SpatialLeiden HVG PCA | SpatialLeiden SVG PCA | SpatialLeiden SVG msPCA |
| --- | --- | --- | --- | --- | --- | --- | --- | --- |
| BayesSpace |  | 0.0011 | 0.0011 | 0.0011 | 0.042 | 0.11 | 0.16 | 0.31 |
| Leiden HVG PCA | 0.0011 |  | 0.057 | 0.0017 | 0.0017 | 0.0011 | 0.0011 | 0.0011 |
| Leiden SVG PCA | 0.0011 | 0.057 |  | 0.0017 | 0.0017 | 0.0011 | 0.0011 | 0.0011 |
| Leiden SVG msPCA | 0.0011 | 0.0017 | 0.0017 |  | 0.027 | 0.0011 | 0.0011 | 0.0011 |
| SpaGCN | 0.042 | 0.0017 | 0.0017 | 0.027 |  | 0.16 | 0.15 | 0.057 |
| SpatialLeiden HVG PCA | 0.11 | 0.0011 | 0.0011 | 0.0011 | 0.16 |  | 0.52 | 0.11 |
| SpatialLeiden SVG PCA | 0.16 | 0.0011 | 0.0011 | 0.0011 | 0.15 | 0.52 |  | 0.057 |
| SpatialLeiden SVG msPCA | 0.31 | 0.0011 | 0.0011 | 0.0011 | 0.057 | 0.11 | 0.057 |  |

**Table S3:** Adjusted p-values for differences in NMI for the Stereo-seq datasets (two-sided Wilcoxon signed-rank test and Benjamini-Hochberg procedure).

| vs | n | SpatialLeiden (HVG PCA square) | | SpatialLeiden (SVG PCA square) | | SpatialLeiden (SVG msPCA square) | |
| --- | --- | --- | --- | --- | --- | --- | --- |
|  |  | **p-value** | **p BH-adjusted** | **p-value** | **p BH-adjusted** | **p-value** | **p BH-adjusted** |
| BASS | 8 | 0.00781 | 0.0125 | 0.00781 | 0.0104 | 0.00781 | 0.0125 |
| DeepST | 6 | 0.0312 | 0.0357 | 0.0312 | 0.0357 | 0.0312 | 0.0357 |
| SCAN-IT | 9 | 0.00391 | 0.0104 | 0.00391 | 0.00781 | 0.00391 | 0.00781 |
| SEDR | 8 | 0.00781 | 0.0125 | 0.00781 | 0.0104 | 0.0156 | 0.0208 |
| SpaGCN_without | 9 | 0.0195 | 0.026 | 0.00391 | 0.00781 | 0.00391 | 0.00781 |
| SpaceFlow | 9 | 0.00391 | 0.0104 | 0.00391 | 0.00781 | 0.00391 | 0.00781 |
| conST_nopre | 5 | 0.0625 | 0.0625 | 0.0625 | 0.0625 | 0.0625 | 0.0625 |
| leiden | 9 | 0.00391 | 0.0104 | 0.00391 | 0.00781 | 0.00391 | 0.00781 |

**Table S4:** SpaceHack v2.0 participants and their CRediT attribution

| **Name** | **Email** | **ORCiD** | **Affiliation** | **CRediT** |
| --- | --- | --- | --- | --- |
| Ahmed Mahfouz | a.mahfouz@lumc.nl | 0000-0001-8601-2149 | Department of Human Genetics, Leiden University Medical Center, Einthovenweg 20, 2333 ZC Leiden, Netherlands | Conceptualization |
| Alexander Kanitz | alexander.kanitz@unibas.ch | 0000-0002-3468-0652 | Biozentrum, University of Basel, Spitalstrasse 41, 4056 Basel, Switzerland | Conceptualization, Data Curation, Investigation |
| Brian Long | brianl@alleninstitute.org | 0000-0002-7793-5969 | Allen Institute for Brain Science, 615 Westlake Ave N, Seattle, WA, USA 98109 | Conceptualization, Writing – review & editing |
| Fadhl Alakwaa | alakwaaf@umich.edu | 0000-0001-5349-7960 | Department of Internal Medicine, Division of Nephrology, University of Michigan, Ann Arbor, Michigan, USA | Conceptualization |
| Florian Heyl | florian.heyl@dkfz-heidelberg.de | 0000-0002-3651-5685 | German Cancer Research Center (DKFZ), Division of Computational Genomics and Systems Genetics and German Human Genome-Phenome Archive (GHGA), Im Neuenheimer Feld 280, 69120 Heidelberg, Germany | Conceptualization, Writing – review & editing |
| Francesca Antonella Luongo | fluongo@student.ethz.ch | 0009-0005-6475-9029 | ETHZ, Rämistrasse 101, 8092 Zürich / CSEM Hegenheimermattweg 167A, 4123 Allschwil | Writing – review & editing |
| Georgios Gavriilidis | ggeorav@certh.gr | 0000-0003-2575-4354 | Institute of Applied Biosciences, Centre for Research and Technology Hellas, Thessaloniki, Greece | Writing – review & editing |
| Giorgia Moranzoni | gimo@dtu.dk | 0000-0001-8065-6277 | DTU Health Tech, Technical University of Denmark, Ørsteds Plads, Building 345C, 2800, Kgs. Lyngby, Denmark | Writing – review & editing |
| Jieran Sun | jieran.sun@chuv.ch | 0000-0002-7996-3840 | Biomedical Data Science Center, Centre hospitalier universitaire vaudois, Rue du Bugnon 21, 1011 Lausanne | Conceptualization |
| Liya Zaygerman | liya.zaygerman@tum.de | 0009-0005-4947-6223 | Helmholtz Zentrum München, Institute of Computational Biology, Ingolstädter Landstraße 1 · D-85764 Neuherberg | Conceptualization |
| Lucie Pfeiferova | lucie.pfeiferova@img.cas.cz | 0000-0003-1089-0329 | Laboratory of Genomics and Bioinformatics, Institute of Molecular Genetics of the Czech Academy of Sciences, 142 20, Prague 4, Czech Republic. | Writing – review & editing |
| Mark D. Robinson | mark.robinson@mls.uzh.ch | 0000-0002-3048-5518 | SIB Swiss Institute of Bioinformatics and Department of Molecular Life Sciences, University of Zurich, Winterthurerstrasse 190, 8057 Zurich, Switzerland | Conceptualization |
| Meghan A. Turner | meghan.turner@alleninstitute.org | 0000-0003-2451-5036 | Allen Institute for Brain Science, 615 Westlake Ave N, Seattle, WA 98109 | Writing – review & editing |
| Peiying Cai | peiying.cai@uzh.ch | 0009-0001-9229-2244 | Department of Molecular Life Sciences, University of Zurich, Winterthurerstrasse 190, 8057 Zurich, Switzerland | Conceptualization |
| Rasool Saghaleyni | rasools@chalmers.se | 0000-0003-0956-039X | Department of Biology and Biological Engineering, Chalmers University of Technology, 412 96 Gothenburg, Sweden | Writing – review & editing |
| Sikander Hayat | shayat@ukaachen.de | 0000-0001-5919-8371 | University Hospital RWTH Aachen, Pauwelsstraße 30, Aachen, Germany | Writing – review & editing |
| Søren Helweg Dam | sohdam@dtu.dk | 0000-0003-0755-0016 | DTU Health Tech, Technical University of Denmark, Ørsteds Plads, Building 345C, 2800, Kgs. Lyngby, Denmark | Writing – review & editing |
| Sven Twardziok | sven.twardziok@bih-charite.de | 0000-0002-0326-5704 | Berlin Institute of Health at Charité Berlin, Anna-Louisa-Karsch-Straße 2, 10178 Berlin | Resources |
| Teresa Zulueta-Coarasa | teresaz@ebi.ac.uk | 0000-0002-0456-6912 | European Molecular Biology Laboratory, European Bioinformatics Institute, Hinxton, United Kingdom | Writing – review & editing |
